# The development of prospective memory across adolescence: an event-related potential analysis

**DOI:** 10.3389/fnhum.2015.00362

**Published:** 2015-06-22

**Authors:** Candice Bowman, Tim Cutmore, David Shum

**Affiliations:** School of Applied Psychology, Behavioural Basis of Health, Griffith Health Institute, Griffith UniversityBrisbane, QLD, Australia

**Keywords:** prospective memory, adolescence, ERPs, executive functioning, cognitive development

## Abstract

Prospective memory (PM) is an important cognitive function vital for day-to-day functioning. Although there has been extensive research into the decline of PM in older adulthood, little is known about its developmental trajectory throughout adolescence, a time of important brain maturation. In the present study, the development of PM was examined in 85 participants across the following groups: 12 to 13-year-olds (*n* = 19), 14 to 15-year-olds (*n* = 21), 16 to 17-year-olds (*n* = 19), and 18 to 19-year-olds (*n* = 26). A 30-cue (30 min) event-based PM task (with font-color stimuli as PM cues and a lexical-decision task as the ongoing task) was used while recording Event-Related Potentials (ERPs). The well-established neural correlates of PM, the N300 and parietal positivity, were examined across the age groups. In addition, hierarchical multiple regressions were used to examine the unique contribution of executive functioning measures (viz., the Self-Ordered Pointing Task [SOPT], the Stroop task, and Trail Making Test [TMT]) on the ERP components of PM (after controlling for age). First, the established components of ERPs associated with prospective remembering (i.e., N300 and parietal positivity) were detected for each age group. Second, although there were no significant age- group differences on the amplitude of the N300, the amplitude of the parietal positivity was found to be different between the 12 to 13-year-olds and 18 to 19-year-olds (viz., the 12 to 13-year-olds had the highest amplitude). Third, for the contribution of executive functioning measures on the amplitude of the ERP components of PM, the regression on the N300 was not significant, however, the SOPT beta weights were significant predictors of the amplitude of the parietal positivity. This relationship was found to be specific for the central and right electrode region. These findings are discussed within the context of brain development and executive functioning along with particular task demands, which may contribute to age-related PM differences across adolescence. Moreover, the findings suggest that cognitive processes associated with parietal positivity may continue to develop across adolescence.

## Introduction

A fundamental aspect of attaining independence and autonomy from childhood to adulthood is the ability to carryout future intentions at the appropriate moment in time, such as remembering to take one’s lunch to school or remembering to return a library book on time. This process of remembering to remember is referred to as prospective memory (PM; McFarland and Glisky, 2009) and has been considered the “cornerstone of cognitive development” (Zimmermann and Meier, 2006, p. 2040). To date, there has been extensive research into the nature of PM decline in older adulthood (for a review see Henry et al., 2004) and a growing interest into the development of PM from infancy to late childhood (for a review see Kvavilashvili et al., 2008). What is still unclear, however, is the extent to which PM develops throughout adolescence, a period in which significant changes in the brain take place, especially in the prefrontal lobes (for a review see Paus, 2005).

Presently, there is a growing consensus that the prefrontal lobes play an important part in supporting prospective remembering (West, 1996; McDaniel et al., 1999). In particular, it has been argued that PM tasks generally rely on self-initiated and conscious cognitive processes that are reliant on the prefrontal lobes (McFarland and Glisky, 2009). Moreover, successful prospective remembering is assumed to be supported by two distinct components, the prospective component, which supports the detection of cues in the environment, and the retrospective component, which supports the retrieval of the previously formed intention from memory (Einstein and McDaniel, 1990). Despite PM having a retrospective component, PM tasks differ from retrospective memory (RM) tasks because PM tasks incorporate a number of underlying cognitive processes that are not required by standard RM tasks (McFarland and Glisky, 2009). For instance, one must first form an intention (e.g., bring a signed permission slip to school), hold it in mind while working on other ongoing activities (e.g., household chores or watching TV), monitor the environment for PM cues so to initiate the action at the right time (e.g., see the signed permission slip stuck on the fridge), and execute that previously formed intention, such as placing the slip in one’s schoolbag (Kliegel et al., 2002). These underlying processes have been linked to the prefrontal system (McDaniel et al., 1999; Burgess et al., 2003).

One rationale for the improvement of PM across adolescence pertains to the age-related efficiency of executive functioning across this period (Wang et al., 2011). In particular, PM has been found to be associated with executive functions served by the prefrontal cortex, including working memory, inhibition, and task-switching (McDaniel et al., 1999). More specifically, working memory may be associated with the ability to hold in mind multiple task sets; inhibition may be associated with the ability to interrupt and inhibit the ongoing task (when the cue is detected); and task-switching may be associated with the ability to switch flexibly from the ongoing task to the PM task so to initiate the PM action (Rose et al., 2010; Kliegel et al., 2011; Schnitzspahn et al., 2013). In line with the significant brain changes that occur during adolescence (e.g., Paus et al., 2001; Giedd, 2004), these aspects of executive functioning (in particular working memory and task-switching) have been found to be still developing across adolescence (e.g., Anderson et al., 2001; Huizinga et al., 2006). Moreover, a number of studies have found that certain executive functioning measures (i.e., the Stroop task, Trial Making Task [TMT], and Self-Ordered Pointing Task [SOPT]) to predict PM performance in children and adolescents, after controlling for age (e.g., Altgassen et al., 2014; in the interruption condition, Shum et al., 2008; in the high-demand condition, Ward et al., 2005). Therefore, it has been proposed that the improvement of executive functioning may underlie the development of PM from childhood to adulthood (West, 1996; Kliegel et al., 2000; Wang et al., 2011).

Despite the strong rationale for the improvement of PM across adolescence, research with this age range is particularly limited, and studies that have examined PM performance in adolescents have yielded inconsistent findings. For instance, although some studies have suggested that PM performance in adolescents is similar to that of young adults (Ward et al., 2005; Zimmermann and Meier, 2006), other studies have suggested that PM performance in adolescents is worse than that of young adults (Wang et al., 2006; Zöllig et al., 2007). Differences in the findings of studies may have to do with specific characteristics of the PM task, such as the demand or difficulty of the ongoing task (Kvavilashvili et al., 2008), the type of PM cue used (e.g., focal or non-focal cues, Wang et al., 2011), or the frequency of the PM cues (Wilson et al., 2013). In addition, age-related improvements in nonexecutive processes, such as processing speed or the use of cognitive strategies, may also contribute to the likelihood of whether or not age-related differences on a particular cognitive function would emerge (Luna et al., 2004; Segalowitz and Davies, 2004; Davidson et al., 2006). In fact, Segalowitz and Davies (2004) argued that adolescents might not use the same cognitive strategies as young adults. Specifically, they argued that unlike young adults, children and adolescents do not have access to a “highly integrated prefrontal cortex” (p. 130); therefore, they may have to recruit different brain regions to perform the same task (e.g., Zöllig et al., 2007). Taken together, the developmental trajectory of PM across adolescence is still unclear.

More recently, it has been proposed that processes underling the prospective component of PM (i.e., monitoring for PM cues) may contribute to age-related differences in PM across adolescence (Wang et al., 2011; Altgassen et al., 2014). Specifically, it has been argued that PM performance in adolescents should be poorer than that of young adults on tasks that impose greater demand on executive resources needed to monitor for the PM cue (e.g., tasks that utilize nonfocal cues, Wang et al., 2011). The rationale for this proposal is in largely based on the predictions of the Multiprocess Theory (MPT; McDaniel and Einstein, 2000) and the Preparatory Attentional and Memory Processes (PAM) theory (Smith, 2003). Both theories have proposed that PM retrieval (under some conditions) may require a high level of strategic monitoring processes to detect the PM cue. In particular, nonfocal PM cues (i.e., PM cues that do not have any defining features of the ongoing task) are assumed to require a greater degree of attentional processes to monitor for the PM cue (see Einstein et al., 2005). For this reason, PM performance should be poorer in adolescents than that of young adults on tasks that load heavily on executive control processes as executive functions associated with these processes (i.e., working memory, inhibition, and task-switching) are still developing in adolescence (e.g., Anderson et al., 2001; Huizinga et al., 2006). In support of this, a number of studies have shown adolescents to have poorer PM performance on tasks that have utilized nonfocal cues (e.g., Zöllig et al., 2007; Wang et al., 2011; Altgassen et al., 2014). In contrast, some studies (particularly Event-Related potential [ERP] studies) have suggested that processes underlying the retrospective component may contribute to age-related differences in PM across adolescence, whereas processes underlying the prospective component of PM may already be developed by adolescence (Zöllig et al., 2007; Smith et al., 2010; Mattli et al., 2011).

During the last decade, significant advances have been made in understanding the neurocognitive processes that underlie PM with the use of ERPs (see West, 2008, 2011). Studies that have used ERPs to examine PM have allowed researchers to identify the specific time course of neural correlates of prospective remembering, including those associated with the prospective component of PM (N300), and those associated with the retrospective component of PM (parietal positivity; West, 2008, 2011). The N300 reflects a negativity over the occipital-parietal region of the scalp. It begins around 200 ms after stimulus onset and has a maximal amplitude around 300–500 ms after stimulus onset (West, 2011). It reflects a greater negativity for PM cues than for ongoing trials (West et al., 2007; West, 2011), and may be similar for PM misses and ongoing trials (West et al., 2007; Mattli et al., 2011). Following the manifestation of the N300 is the parietal positivity, which is a sustained positivity around the parietal region of the scalp. The parietal positivity begins around 400 ms after stimulus onset and lasts until around 1200 ms after stimulus onset. Like the N300, the parietal positivity exhibits greater amplitude for PM cues than for ongoing trials and for PM misses (West et al., 2001; West, 2011). Although the parietal positivity has been generally assumed to be associated processes underlying the retrospective component of PM (Zöllig et al., 2007; West, 2008), it may actually encompass processes associated with both the prospective and retrospective components of PM. In particular, the parietal positivity is believed to reflect three distinct components of the ERPs (West, 2011). These include the P3b, which is associated with the detection of low probable events (Luck, 2005), the old-new effect, which is associated with the retrieval of an intention from memory (West and Krompinger, 2005), and the prospective positivity, which may be associated with the configuration of the PM task set (West, 2011). The old-new effect is believed to share processes with both the retrospective component of PM (i.e., retrieval of PM intention from memory) and explicit episodic memory (recognition and cued recall; West and Krompinger, 2005). However, the P3b and the prospective positivity may reflect processes associated with the detection of PM cues and the configuration of the PM task set respectively (see Mattli et al., 2011; West, 2011). The ERP correlates of PM have been examined across the lifespan from childhood to later adulthood (Zöllig et al., 2007; Mattli et al., 2011).

Zöllig et al. (2007) examined the development of the neural correlates of PM (i.e., the N300 and parietal positivity) across the lifespan. Fourteen adolescents (*M* = 12.8 years, *SD* = 0.6 years), 14 young adults (*M* = 22.5 years, *SD* = 1.4 years), and 14 older adults (*M* = 70.1 years, *SD* = 5.5 years) were included in this study. For the ongoing task, participants were required to complete a semantic relatedness task. For the PM task, participants had to first form the intention (i.e., intention formation trials) by remembering the color and the letter of a presented letter string (i.e., letters “cccc” or “vvvv” in the color magenta or gray). For PM trials, participants were required to press the target key (e.g., “c” or “v”) whenever the PM cue was presented (e.g., a word pair presented in magenta or gray). PM inhibit trials were also part of the task; and participants were instructed to ignore the PM cue and just make a semantic judgment. First, the findings of this study showed that PM failures in adolescents might have resulted from problems associated with the retrospective component of PM (i.e., PM errors due to not remembering the intended action). In contrast, they found no significant differences in PM misses (i.e., PM errors due to the ongoing task response being made or timeouts) between adolescents and young adults. Second, the amplitude of the N300 was similar across the age groups for PM trials. The amplitude of the parietal positivity, however, was found to increase from adolescence to adulthood, and then decrease from adulthood to older adulthood. Zöllig et al. (2007) concluded that different underlying processes may have contributed to the development of PM across the lifespan. Moreover, they argued that processes underlying the prospective component of PM might already be fully developed by adolescence, whereas processes underlying the retrospective component may continue to develop until adulthood (see also Mattli et al., 2011).

Taken together, the specific processes (viz., prospective or retrospective) that contribute to PM development in adolescence is still unclear as both behavioral and ERP studies have yielded inconsistent findings. Hence, the aim of this study was to examine the neural correlates of PM across adolescence. First, ERPs were used in this study in order to examine the neural correlates associated with the detection of PM cues (i.e., N300) and the retrieval of an intention from memory (i.e., parietal positivity) across adolescence. This may help give further insight into the underlying processes that contribute to PM development across adolescence (West, 2011). The study also included a wide age-range of participants (12 to 19-years). By using several groups across a wider age-range of adolescents, a more fine-grained analysis into the protracted progression of PM across adolescence can be implemented. Finally, the PM task utilized nonfocal PM cues in order to increase the likelihood of finding age-related differences in PM across adolescence (McDaniel and Einstein, 2000; Wang et al., 2011).

In sum, the aim of the current study was to examine the neural correlates of PM (i.e., the N300 and parietal positivity) across adolescence. First, if processes underlying the prospective component of PM continue to develop across adolescence, then the amplitude of the N300 should reveal an age-related difference (Wang et al., 2011). By comparison, if processes underlying the retrospective component of PM continue to develop across adolescence, then the amplitude of the parietal positivity should reveal an age-related difference (Zöllig et al., 2007). Finally, the contribution of executive functioning (viz., working memory, inhibition, and task-switching) on the amplitude of the N300 and parietal positivity was examined. A number of studies have previously demonstrated the link between executive functions and behavioral measures of PM performance (e.g., McDaniel et al., 1999; Kliegel et al., 2008). More importantly, if executive control processes contribute to successful prospective remembering in adolescence (Wang et al., 2011), then the association between executive functioning and PM should be reflected in the ERPs. Specifically, if processes underlying the prospective component of PM continue to develop across adolescence, then executive functioning may have a greater contribution to the amplitude of the N300 (i.e., prospective component of PM), particularly if nonfocal PM tasks place a higher demand on executive control processes needed to detect PM cues (e.g., Ward et al., 2005; Shum et al., 2008; Altgassen et al., 2014). Conversely, if age-related differences in the parietal positivity contribute to PM development in adolescence (Zöllig et al., 2007), then it is unclear as to the extent to which executive functioning would contribute to the amplitude of parietal positivity (West, 2011). Specifically, if the parietal positivity encompasses processes that also support the detection of PM cues, then executive functioning may also contribute to this modulation. The study was exploratory in nature and was the first to explore how executive functions may contribute to the neurocognitive processes that underlie PM in adolescence.

## Methods

### Participants

Participants in this study were 93 adolescents and young adults. Sixty-five adolescents (22 males and 43 females) between the ages of 12 and 17 years were recruited from the Brisbane Metropolitan area via local newspaper advertisement, participants known to the researcher and children of Griffith University staff members. Adolescents were divided into three age groups: Group 1 (12 to 13-year-olds, *n* = 22), Group 2 (14 to 15-year-olds, *n* = 23), and Group 3 (16 to 17-year-olds, *n* = 20). In addition, Group 4 (18 to 19-year-olds) comprised 28 young adults (10 males and 18 females) and they were recruited from the Griffith University first year participant pool. Participants who had a history of brain damage, sensory deficits (e.g., vision loss) or had a diagnosis of a learning disability, psychiatric or behavioral disorder were excluded from the study. Adolescents were compensated with one movie ticket, and one candy bar voucher for their time. Young adults were given course credit for their participation.

Three participants had to be excluded from the study. Two participants obtained low scores (below 1 *SD* for their age-group) on one of the two subtests (viz., verbal) of the Wechsler Abbreviated Scale of Intelligence (WASI; Psychological Corporation, 1999) and one participant was not available to complete all parts of the testing. Table 1 shows the final sample sizes, mean age, and estimated IQ (viz., two subtest version of the WASI: verbal and matrix reasoning) for the current study. A one-way ANOVA revealed no significant difference between the four age groups on mean estimated IQ score, *F*_(3,85)_ = 0.80, *p* = 0.50.

**Table 1:** **Final sample sizes, mean age and estimated IQ score (two subtest version of the WASI) for each of the age groups (standard deviations in parentheses)**.

Age group	*N*	Age	Estimated IQ
Group 1 (12 to 13-year-olds)	22	12.64 (0.51)	110.14 (10.55)
Group 2 (14 to 15-year-olds)	22	14.58 (0.51)	105.55 (10.81)
Group 3 (16 to 17-year-olds)	19	16.53 (0.57)	106.61 (9.10)
Group 4 (18 to 19-year-olds)	27	18.47 (0.51)	107.37 (10.34)

### Ethical Clearance

The current study has received ethical approval from the Griffith University Human Research Ethics Committee (Reference No GU Ref No: PSY/A8/10/HREC). The committee is constituted and operates in accordance with the National Statement on Ethical Conduct in Human Research (2007).

### Materials

#### PM

**Figure 1:**
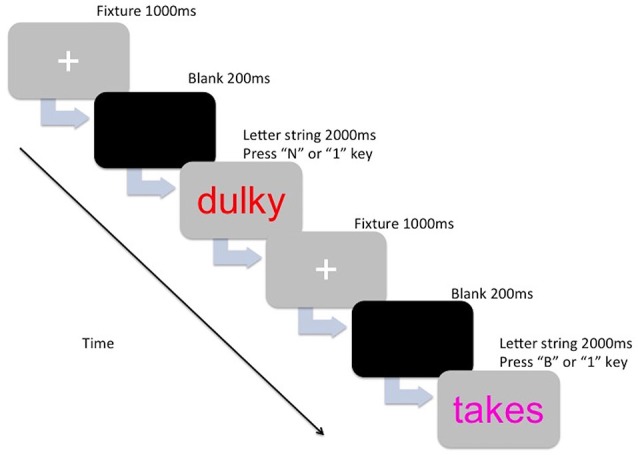
**The presentation for one trial for either the ongoing component (lexical decision—Press “B” or N” key) or the prospective memory (PM) component (Press “1” key)**.

The stimuli of the experimental task consisted of colored letter strings (5–7 characters long) that were either English words or non-words. The letter strings were presented in the center of the computer screen (Dell 22-inch computer monitor) in lowercase Arial 48 point font, in the colors magenta, blue, red, and orange. The words and non-words were selected from The English Lexicon Project (Balota et al., 2007). Words were high frequency words and had a high mean accuracy (i.e., 0.80 and above). Non-word selection was based on orthographic neighborhood (four to six orthographic neighbors), and also had a high mean accuracy (for a review on the lexical statistics see Balota et al., 2007). The letter strings used for the practice trials were not repeated in the main trials. The PM cues consisted of a target color (magenta, blue, red, or orange) and each participant was assigned a color to be used as the PM cue. The other three colors were assigned to the ongoing task letter strings (e.g., if a participant was allocated the color magenta for the PM cue, then the ongoing letter strings would have been presented in blue, red, and orange for that participant). Color assignment was counter-balanced across age groups. For the *ongoing task component*, participants were instructed to indicate whether the letter string made a word (press “B” key) or a non-word (press “N” key). For the *PM task component*, participants were instructed to withhold the ongoing task response and press a target key (press the “1” key) whenever they encountered the PM cue (i.e., the target color assigned to them). Figure 1 shows the breakdown of one trial.

A pilot was conducted with six participants (aged between 11 to 21-years) to help determine ongoing task difficulty, presentation speed of the letter strings, the number of PM cues to use for the PM task, and total task running time. All six participants achieved at least 90% accuracy on the ongoing task.

The PM task consisted of a total of 600 trials (30 blocks of 20 trials) of which 570 trials were ongoing trials and 30 were PM trials. The PM cues were randomly allocated between trials 10–20 for each block of 20 trials in order to discourage counting or anticipation.^1^ There was a rest period just after every 200th trial (two rest periods in total) where participants were told to rest their eyes and press the space bar button when they were ready to continue. Eighteen practice trials (with one PM cue) were given prior to testing. The task took approximately 30 min to complete.

#### Executive Function Measures

##### Stroop color and word interference test

The Stroop task (Lezak et al., 2004) is a measure of verbal response inhibition and attention. The test is appropriate to use for individuals aged 5–90 years (Golden, 1978; Golden and Freshwater, 2002). The Stroop task comprises three different trials: word-reading, color-naming, and color-word interference. First, the word-reading trial was given and this consists of 100 words “RED”, “GREEN, and “BLUE” printed in black ink on a sheet of paper. Participants were required to read the words as quickly as they could. Following this trial, the color-naming trial was given, and this consists of 100 Xs (i.e., “XXXX”) printed in red, green or blue ink on a sheet of paper. Participants were required to name the ink color as quickly as they could. Finally, the color-word interference trial was given and this consists of a 100 printed words (words: “RED”, “GREEN” and “BLUE”) that were printed in an incongruent color (e.g., the word “RED” printed in blue) on a sheet of paper. Participants were required to name the ink colors of the words as quickly as they could. All items were presented in random order on the sheet of paper (five columns with 20 items each). Participants were given a time limit of 45 s for each trial. The Stroop task took approximately 5 min to administer and the number of correct responses was summed for each trial. Interference scores were computed by subtracting the number of correct responses on the color-word interference trial from the number of correct responses predicted using the formula: (C × W)/(C + W) (see Golden and Freshwater, 2002). The interference score was then used as the dependent measure.

##### Trail Making Test (TMT)

The TMT is a test designed to assess visual tracking and cognitive flexibility (Strauss et al., 2006). The test has two parts, Part A (visual tracking and sequence ability) and Part B (cognitive flexibility). For Part A, the worksheet consisted of 25 circled numbers. The circled numbers were scattered across the page. Participants were required to draw lines connecting the 25 circled numbers in consecutive ascending order, as quickly as possible without lifting the pen from paper. For Part B, the worksheet consisted of 25 circled numbers and letters. The circled numbers and letters were also scattered across the page. Participants were required to draw lines connecting the circled numbers and circled letters in ascending order, alternating between them (e.g., 1-A-2-B-3-C etc.). Performance was assessed by the time taken (in seconds) to complete each trial correctly (Strauss et al., 2006). The switching score computed was the time taken to complete Trail B minus the time taken to complete Trail A.

##### Self-Ordered Pointing Task (SOPT)

The SOPT is a measure of visual working memory, based on the representational drawings task (Lezak et al., 2004). A computerized version of this test was used (Petrides and Milner, 1982). The task was divided into four blocks: a 6-picture block, an 8-picture block, a 10-picture block, and a 12-picture block. Colored pictures were presented on a computer screen. There were three trials for each picture block (a total of 12 trials in total) and participants were required to click on a different picture on each screen. The pictures would automatically reshuffle into new positions as soon as the participant made a response. Specifically, the pictures reshuffled six times for the 6-picture block, eight times for the 8-picture block, and so on. They were instructed not to click on the same picture twice or the same location multiple times. Pictures that were pointed to more than once, in the same trial (for each block), were counted as errors. The number of errors for each picture block was summed and the total amount of errors for the test was computed for each participant.

### Electrophysiological Recording and Analysis

#### Recoding

Electrophysiological data was recorded using the Active Two BioSemi system (version 6.05, 2010) from an array of 64 channels at a sampling rate of 1024 Hz. The BioSemi system uses 24-bit Ag/AgCl active electrodes with built in digital amplifiers (band-pass DC 206 Hz). Vertical eye movements were recorded from electrodes placed below and above the left eye. Horizontal eye movements were recorded by placing electrodes on the outer canthus of each eye. During the recording electrodes were re-referenced to an average reference (for guidelines see Picton et al., 2000).

#### Processing

All bioelectrical signals were digitized on a laboratory computer using the BioSemi Active View software. Offline analysis was performed using BESA (version 5.3.6, GmbH, Germany). The BESA artifact correction algorithm was used to reduce the influence of contaminating ocular sources to the EEG. After artifact correction, a bandpass filter of 0.1 Hz (6 db/oct; forward) to 30 Hz (24 db/oct; 0 phase) and notch filter of 50 Hz were applied. ERP analysis epochs were extracted off-line and included a 200 ms of pre-stimulus activity and 1200 ms of post-stimulus activity (p. 3312, Zöllig et al., 2007). Trials containing residual artifact were rejected before averaging (cut-off ±120 μV). For each participant, the average ERPs were computed for correct responses for the ongoing trials and PM trials (West and Krompinger, 2005) and the N300 and the parietal positivity were quantified using area amplitude in appropriate time windows.

#### Analysis of Mean Amplitude

Differences in mean amplitude between the four age groups were analyzed using a mixed-factorial ANOVA. The selection of electrodes and latency windows were guided by previous research (Zöllig et al., 2007) and a visual inspection of the grand averages. The N300 was quantified as mean amplitude between 200 and 300 ms after stimulus onset and included data for five electrodes: P7, O1, Oz, O2, P8 (Zöllig et al., 2007). For the analyses, electrodes within one region were collapsed to get a mean activity. The left electrode region included the data for the P7 and O1 electrodes, while the right electrode region included the data for the O2 and P8 electrodes (Mattli et al., 2011).

For the parietal positivity, the mean amplitude was quantified between 450–750 ms after stimulus onset and included data of electrodes: CP3, P3, CPz, Pz, CP4 and P4 (Zöllig et al., 2007). For the analyses, electrodes within one region were also collapsed to get a mean activity. The left electrode region included the data for the CP3 and P3 electrodes and the right electrode region included the data for the CP4 and P4 electrodes (Mattli et al., 2011). The central region was also included for the analysis of the parietal positivity and included the data for the CPz and Pz electrodes.

### Procedure

Participants were tested across two sessions (at least 1 week apart). Half of them completed the behavioral testing session first (which included the completion of executive functioning measures) and the remaining half completed the electroencephalograph (EEG) recording session first (EEG recording for the PM task).^2^ Testing order was counter-balanced across age groups. All participants were tested individually at the Neuropsychological Lab at Griffith University, Mt Gravatt campus. Signed parental/guardian consent was obtained at the first session and participants were given an overview of the procedure for that session. The behavioral session went for approximately 1 h. For the EEG session, participants were fitted with an EEG cap (small, med or large size). After a good EEG signal was obtained, the instructions for the PM task on the computer screen were read out loud to the participants. Participants were encouraged to ask any questions that they may have. Overall, this session lasted for approximately 90 min in total. At the end of the second session, participants were debriefed about the study.

## Results

### Data Screening

Prior to analysis, missing values, normality of the distributions and outliers were checked separately for each dependent variable across each age group. One person’s score (12 to 13-year-old) was excluded from the Stroop task due to the participant having some confusion with the colors. In addition, the data from five participants were excluded from the PM task (both behavioral data and the EEG recording). Of these one misunderstood the task instructions (12 to 13-year-old), one could not complete the task due to a technical problem with the computer (14 to 15-year-old), and three reported experiencing fatigue during the experiment (two 12 to 13-year-olds, and one young adult). Finally, 10 participants had excessive noise in the EEG (three 12 to 13-year-olds, three 14 to 15-year-olds, two 16 to 17-year-olds, and two 18 to 19-year-olds) and their EEG recording was removed from the analysis. Alpha levels for all statistical tests were set at *p* = 0.05. For pairwise comparisons, a Bonferroni adjustment was used to control for Familywise Type I error rate (Howell, 2013). Greenhouse-Geisser corrected degrees of freedom were also applied if sphericity could not be assumed.

### Performance Accuracy (PM task)

Three different scores for each component of the task (ongoing and PM) were used to compare performance across the age groups. Specifically, percent correct scores, reaction time (RT) for correct scores, and a composite score (viz., a combination of unit-weighted z-scores for percentage correct and RT) were computed for ongoing and PM performance (Anderson et al., 1996; Salthouse and Hedden, 2002). For the composite score, percent correct and RT for correct responses were first converted to z-scores. Following that, RT z-scores were then reversed (1 RT z-score) in order to ensure the consistency of the scale (i.e., higher score equals better performance). Finally the z-scores were summed up to form the composite scale (see jeromyanglim.blogspot.com.au).

It is important to note that the EEG recording revealed that there were a number of responses where the incorrect response on the PM trials was made, followed by the correct PM response. While late PM responses may indicate that the PM task was recalled (see Kvavilashvili, 1998), the behavioral and EEG output classified these responses as PM errors. Therefore, these types of responses were not included in the PM behavioral or ERP analysis. Table 2 shows the means and standard deviations for ongoing task and PM performance for the four age groups.

**Table 2:** **Mean percentage correct, reaction time (RT), and composite score for the ongoing and PM task for each age group (standard deviations in parentheses)**.

Age group	*N*	Percentage correct	RT	Composite score
**Ongoing task**
12 to 13-year-olds	19	93.60 (4.70)	900.89 (163.11)	−1.59 (2.36)
14 to 15-year-olds	21	96.15 (2.67)	768.32 (113.91)	0.13 (1.32)
16 to 17-year-olds	19	96.20 (3.30)	781.63 (122.53)	0.05 (1.45)
18 to 19-year-olds	26	98.00 (1.22)	718.45 (84.74)	1.03 (0.79)
**PM task**
12 to 13-year-olds	19	87.89 (15.36)	955.72 (237.10)	−0.54 (1.39)
14 to 15-year-olds	21	84.60 (16.35)	803.53 (180.78)	−0.09 (1.45)
16 to 17-year-olds	19	85.61 (13.43)	855.04 (252.52)	−0.25 (1.57)
18 to 19-year-olds	26	92.95 (6.95)	779.80 (188.36)	0.64 (0.87)

#### Ongoing Task

Percentage correct was found to be the highest in 18 to 19-year-olds and the lowest in the 12 to 13-year-olds. A one-way ANOVA revealed a significant difference between the four age groups on ongoing task percentage correct, *Welch’s F*_(3,35.92)_ = 8.20, *p* < 0.001, *ω*^2^ = 0.20. Levene’s test indicated unequal variances (*F* = 8.72, *p* < 0.001), so *post hoc* comparison Games-Howell was used. This showed that 12 to 13-year-olds and 14 to 15-year-olds performed significantly worse than 18 to 19-year-olds (*p* < 0.01 and *p* < 0.05 respectively). There were no other significant age group differences.

RT was found to be the highest in the 12 to 13-year-olds. A one-way ANOVA revealed a significant difference between the four age groups on RT for the ongoing task, *F*_(3,84)_ = 8.55, *p* < 0.001, *ω*^2^ = 0.21. *Post hoc* comparison using the Tukey HSD test indicated that the 12 to 13-year-olds performed significantly slower than all the other age groups (14 to 15-year-olds: *p* < 0.01; 16 to 17-year-olds: *p* < 0.05; and 18 to 19-year-olds: *p* < 0.001). There were no other significant age group differences.

Ongoing task composite score was found to be the highest in the 18 to 19-year-olds. A one-way ANOVA revealed a significant difference between the four age groups on ongoing task composite score, *Welch’s F*_(3,38.36)_ = 9.54, *p* < 0.001, *ω*^2^ = 0.23. Levene’s test indicated unequal variances (*F* = 6.26, *p* < 0.01), so *post hoc* comparison Games-Howell was used. This showed that 18 to 19-year-olds had a significantly higher ongoing task composite score than 12 to 13-year-olds (*p* < 0.01) and 14 to 15-year-olds (*p* < 0.05), with trend also for 16 to 17-year-olds (*p* = 0.06). The 14 to 15-year-olds were found to also have a significant higher composite score than the 12 to 13-year-olds (*p* < 0.05). There were no other significant age group differences.

#### PM

Although PM performance was the highest in 18 to 19-year-olds and the lowest in the 14 to 15-year-olds, a one-way ANOVA revealed no significant difference between the four age groups on PM percentage correct, *F*_(3,81)_ = 1.90, *p* = 0.14. In contrast, RT was found to be significantly higher for the 12–13-year-olds, *F*_(3,84)_ = 2.78, *p* < 0.05, *ω*^2^ = 0.06. *Post hoc* comparison using the Tukey HSD test indicated 12 to 13-year-olds performed slower than the 18 to 19-year-olds (*p* < 0.05). There were no other significant age group differences for RT.

Finally, the PM composite score was found to be the lowest in the 12 to 13-year-olds and the highest in the 18 to 19-year-olds. A one-way ANOVA revealed a significant difference between the four age groups on the PM composite score, *F*_(3,84)_ = 3.35, *p* < 0.05, *ω*^2^ = 0.08. *Post hoc* comparison using the Tukey HSD test indicated that the 12 to 13-year-olds had a significantly lower PM composite score than the 18 to 19-year-olds (*p* < 0.05). There were no other significant age group differences.

### Differences in Mean Amplitude

**Figure 2:**
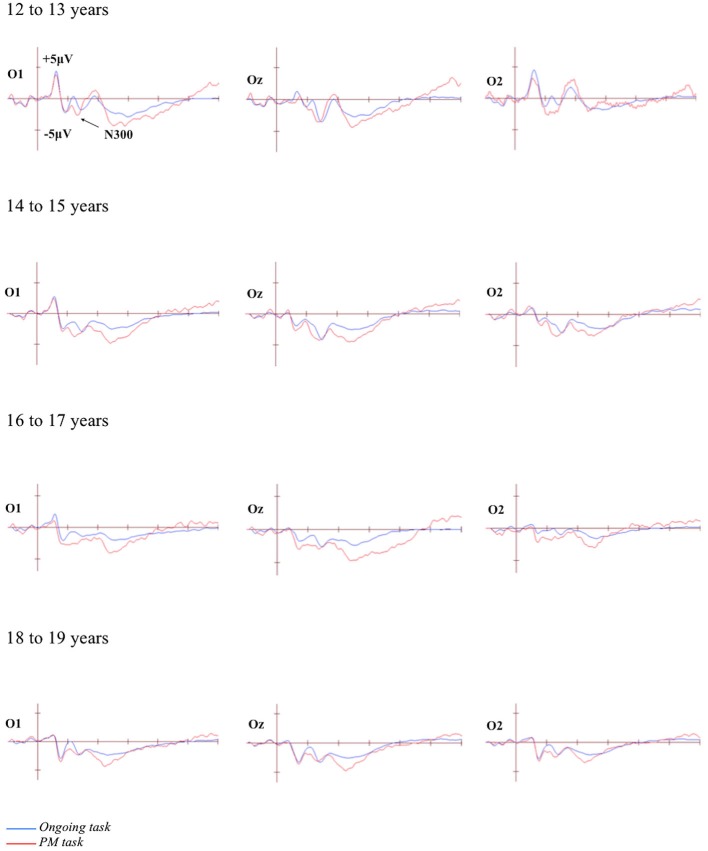
**Grand-averaged event-related potentials (ERPs) at selected electrodes demonstrating the N300 for the ongoing and PM trials for the four age groups**. Horizontal ticks represent 200 ms increments and vertical ticks represent 5 μV.

**Figure 3:**
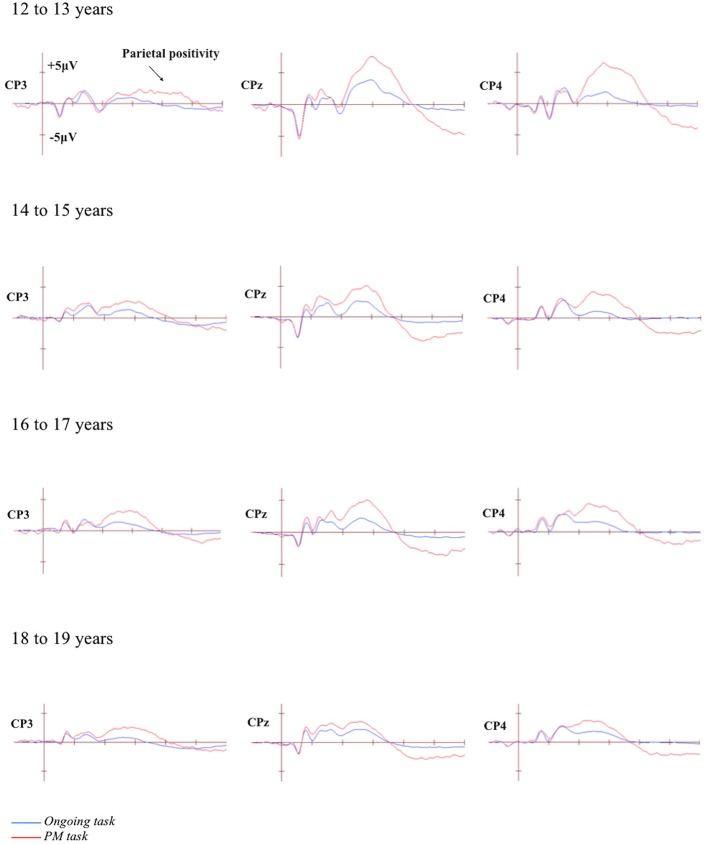
**Grand-averaged event-related potentials (ERPs) at selected electrodes demonstrating the parietal positivity for the ongoing and PM trials for the four age groups**. Horizontal ticks represent 200 ms increments and vertical ticks represent 5 μV.

The grand-averaged ERPs portraying the N300 and parietal positivity for the ongoing and PM trials, for each of the four age groups are presented in Figures 2, 3 respectively. The N300 was observed at the occipital-parietal electrodes and differentiated ongoing task trials from PM trials for each of the age groups. This modulation began around 200 ms after stimulus onset. The parietal positivity was observed at the parietal electrodes between 400 and 800 ms and also differentiated ongoing task trials from PM trials for each of the age groups.

#### N300

The N300 was analyzed using a 4 (Age group: 12 to 13-year-olds, 14 to 15-year-olds, 16 to 17-year-olds, and 18 to 19-year-olds) × 2 (Trial: ongoing and PM) × 2 (Electrode region: left [P7 and O1] and right [P8 and O2]) mixed ANOVA. The main effect of Trial was significant, *F*_(1,71)_ = 74.35, *p* < 0.001, ${\text{η}}_{\rho }^{2}$ = 0.51. This showed that the amplitude was more negative for the PM trials (*M* = −4.32, *SEM* = 0.63) relative to the ongoing task trials (*M* = −1.05, *SEM* = 0.47). The main effect of Electrode region was also significant, *F*_(1,71)_ = 5.94, *p* < 0.05, ${\text{η}}_{\rho }^{2}$ = 0.08. This showed that the amplitude was more negative on the left electrode region (*M* = −3.50, *SEM* = 0.62) than the right electrode region (*M* = −1.87, *SEM* = 0.63). In contrast, the main effect of Age was not significant, *F*_(3,71)_ = 1.94, *p* = 0.13. All the interactions were also not significant: Age group and Trial, *F*_(3,71)_ = 0.38, *p* = 0.77; Age group and Electrode region, *F*_(3,71)_ = 0.67, *p* = 0.57; Trial and Electrode region, *F*_(1,71)_ = 0.51, *p* = 0.47; and the three-way interaction between Age group, Trial and Electrode region, *F*_(3,71)_ = 2.32, *p* = 0.09.

#### Parietal Positivity

The parietal positivity was analyzed using a 4 (Age group: 12 to 13-year-olds, 14 to 15-year-olds, 16 to 17-year-olds, and 18 to 19-year-olds) × 2 (Trial: ongoing and PM) × 3 (Electrode region: left [CP3 and P3], central [CPz and Pz], and right [CP4 and P4]) mixed ANOVA. Mauchly’s test indicated that the assumption of sphericity had been violated for the main effect of Electrode region, *χ*^2^_(2)_ = 8.95, *p* = 0.011, and Trial by Electrode region interaction, *χ*^2^_(2)_ = 7.70, *p* = 0.021. Therefore the Greenhouse-Geisser correction was applied (*ε* = 0.89 for the main effect of Electrode region and 0.91 for Trial by Electrode region interaction). The main effect of Trial was significant, *F*_(1,71)_ = 205.87, *p* < 0.001, ${\text{η}}_{\rho }^{2}$ = 0.74. This showed that the amplitude was more positive for the PM trials (*M* = 13.18, *SEM* = 0.72) than the ongoing task trials (*M* = 5.31, *SEM* = 0.42). The main effect of Electrode region was also significant, *F*_(1.79,126.78)_ = 22.22, *p* < 0.001, ${\text{η}}_{\rho }^{2}$ = 0.24, but the main effect of Age group was not significant, *F*_(3,71)_ = 1.82, *p* = 0.15. This showed that the amplitude was more positive for the right (*M* = 10.89, *SEM* = 0.68) and central electrode region (*M* = 10.54, *SEM* = 0.72) than the left electrode region (*M* = 6.30, *SEM* = 0.66). The interaction between Age group and Electrode region was not significant, *F*_(6,142)_ = 1.50, *p* = 0.18. In contrast, the interaction between Trial and Electrode region was significant, *F*_(1.81, 128.60)_ = 15.37, *p* < 0.001, ${\text{η}}_{\rho }^{2}$ = 0.18, as was the interaction between Age group and Trial, *F*_(3,71)_ = 4.19, *p* < 0.01, ${\text{η}}_{\rho }^{2}$ = 0.15. Finally, the three-way interaction between Age group, Trial, and Electrode region was significant, *F*_(5.43,128.60)_ = 4.38, *p* < 0.01, ${\text{η}}_{\rho }^{2}$ = 0.16.

**Figure 4:**
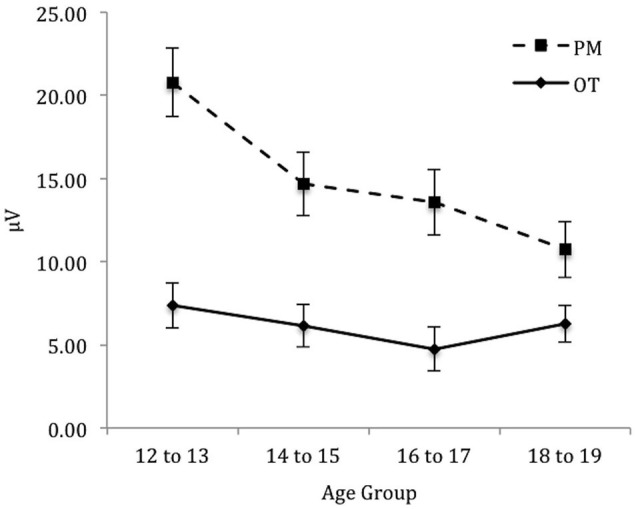
**Mean parietal positivity amplitude by trial (ongoing task [OT] and PM) and age group for the central electrode region**. Error bars represent +/− 1 SEM.

To identify the source of the significant three-way interaction, the analysis was split by electrode region (central, right, and left), and a 2 (Trial) × 4 (Age group) mixed ANOVA was conducted for each electrode region. For the *central* electrode region (see Figure 4) there was a significant main effect of Trial, *F*_(1,71)_ = 133.48, *p* < 0.001, ${\text{η}}_{\rho }^{2}$ = 0.65 and Age group, *F*_(3,71)_ = 2.92, *p* < 0.05, ${\text{η}}_{\rho }^{2}$ = 0.11. The interaction between Trial and Age group was also significant, *F*_(3,71)_ = 6.12, *p* < 0.01, ${\text{η}}_{\rho }^{2}$ = 0.21. The simple effect of analysis on Age was only significant for PM trials, *F*_(3,71)_ = 4.92, *p* < 0.01, ${\text{η}}_{\rho }^{2}$ = 0.17 and not the ongoing task trials, *F*_(3,71)_ = 0.66, *p* = 0.58. Pairwise comparisons showed that the amplitude of the parietal positivity was significantly greater for 12 to 13-year-olds than the 18 to 19-year-olds for the PM trials (*p* < 0.01).

**Figure 5:**
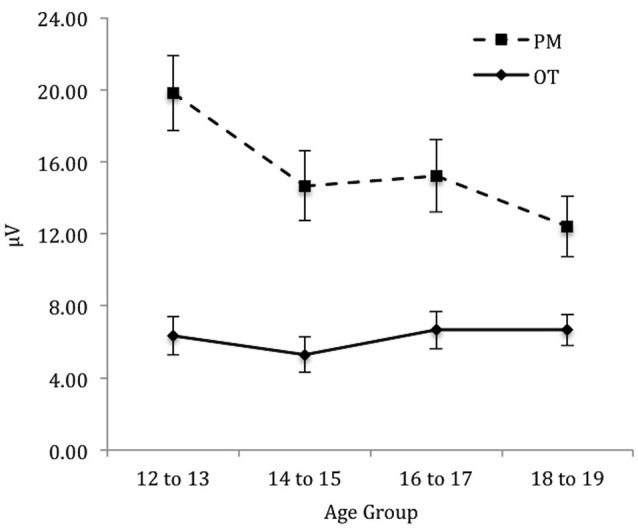
**Mean parietal positivity amplitude by trial (ongoing task [OT] and PM) and age group for the right electrode region**. Error bars represent +/− 1 SEM.

For the *right* electrode region (see Figure 5) there was a significant main effect of Trial, *F*_(1,71)_ = 162.58, *p* < 0.001, ${\text{η}}_{\rho }^{2}$ = 0.70. In contrast, the main effect of Age group was not significant, *F*_(3,71)_ = 1.32, *p* = 0.28. The interaction between Trial and Age group was significant, *F*_(3,71)_ = 4.92, *p* < 0.01, ${\text{η}}_{\rho }^{2}$ = 0.17. The simple effect of Age only showed a marginal significance for PM trials, *F*_(3,71)_ = 2.57, *p* < 0.06, ${\text{η}}_{\rho }^{2}$ = 0.10 and non-significance for the ongoing task trials, *F*_(3,71)_ = 0.44, *p* = 0.73. Pairwise comparisons showed that the amplitude of the parietal positivity was significantly greater for 12 to 13-year-olds than the 18 to 19-year-olds for the PM trials (*p* < 0.05).

**Figure 6:**
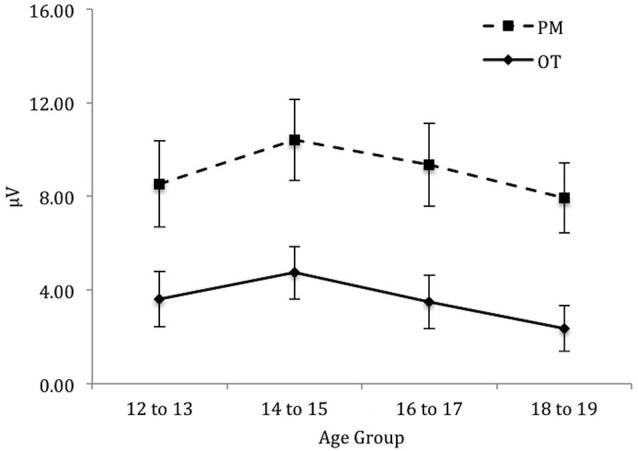
**Mean parietal positivity amplitude by trial (ongoing task [OT] and PM) and age group for the left electrode region**. Error bars represent +/− 1 SEM.

Finally, for the *left* electrode region (see Figure 6) there was a significant main effect of Trial, *F*_(1,71)_ = 88.46, *p* < 0.001, ${\text{η}}_{\rho }^{2}$ = 0.56. In contrast the main effect for Age group was not significant, *F*_(3,71)_ = 0.64, *p* = 0.59. Finally the interaction between Trial and Age group was also not significant, *F*_(3,71)_ = 0.11, *p* = 0.96.

### The Role of Executive Functioning on the Neural Correlates of PM

Hierarchical multiple regressions were conducted to examine the unique contribution of executive functioning on the neural correlates of PM. The independent variables were Age, and the three tests of executive functioning: the Stroop task, TMT (viz., TMTb score) and SOPT. The role of executive functioning on the neural correlates of PM was examined separately for the amplitude of the N300 and parietal positivity. For the parietal positivity, the role of executive functioning on the mean amplitude of the parietal positivity was examined for each electrode region (viz., right, central, and left regions) separately. The regression for the N300 for the overall mean amplitude (i.e., average of P7, O1, Oz, O2, P8) revealed the same results as the region-specific analyses (viz., left and right). Therefore, the role of executive functioning on the overall mean amplitude of the N300 was reported.

#### Data Screening

Prior to analysis, an examination of the assumptions for multivariate analysis (i.e., normality, linearity, multicollinearity, and homoscedasticity) was conducted. With the use of a *p* < 0.001 criterion for Mahalanobis distance, one case was identified as an outlier on the predictor variable (viz., SOPT score). The regressions were conducted with and without this outlier to determine its influence (Field, 2013). The results were the same with and without this outlier. Therefore, it was retained in the analyses in order to maintain generalisability (Tabachnick and Fidell, 2013).

#### N300

Table 3 summarizes the results of the hierarchical regression of executive functioning measures on the amplitude of N300. Age was entered at step 1, explaining 1.5% of the variance in the amplitude of N300. At this step, *R*^2^ = 0.02 was not statistically significant, *F*_(1,71)_ = 1.05, *p* = 0.31. After the entry of the three tests of executive functioning (Stroop, TMT, and SOPT) at step 2, the total variance explained by the model as a whole was 2.1%, *F*_(4,68)_ = 0.37, *p* = 0.83, The *R*^2^ change = 0.01 was not statistically significant, *F* change (3,68) = 0.15, *p* = 0.93.

**Table 3:** **Hierarchical Regression of executive functioning measures on the mean amplitude of the N300**.

Step	Variable	*r*	*B*	*β*
1	Age	−0.12	−0.70	−0.12
2	Stroop	0.03	0.05	0.03
	TMT	0.02	−0.02	−0.02
	SOPT	−0.05	−0.20	−0.08
			*R* = 0.15	*R*^2^ = 0.02

#### Parietal Positivity

Tables 4–6 summarizes the results of the hierarchical regression of executive functioning measures on the amplitude of the parietal positivity for each electrode region.

**Table 4:** **Hierarchical Regression of executive functioning measures on the mean amplitude of the parietal positivity (central region)**.

Step	Variable	*r*	*B*	*β*
1	Age	−0.41**	−1.55	−0.41**
2	Stroop	−0.06	−0.06	−0.05
	TMT	0.20*	0.03	0.07
	SOPT	−0.15	−0.39	−0.22*
			*R* = 0.47**	*R*^2^ = 0.22**

*p < 0.05, **p < 0.01.

**Table 5:** **Hierarchical Regression of executive functioning measures on the mean amplitude of the parietal positivity (right region)**.

Step	Variable	*r*	*B*	*β*
1	Age	−0.26*	−0.98	−0.26*
2	Stroop	0.13	0.16	0.15
	TMT	0.11	0.02	0.03
	SOPT	−0.22*	−0.48	−0.29*
			*R* = 0.41*	*R*^2^ = 0.16*

*p < 0.05.

**Table 6:** **Hierarchical Regression of executive functioning measures on the mean amplitude of parietal positivity (left region)**.

Step	Variable	*r*	*B*	*β*
1	Age	−0.03	−0.09	−0.03
2	Stroop	−0.16	−0.14	−0.16
	TMT	0.02	−0.00	−0.01
	SOPT	−0.05	−0.07	−0.05
			*R* = 0.17	*R*^2^ = 0.03

##### Central electrode region

Age was entered at step 1, explaining 16.5% of the variance in the amplitude of the parietal positivity. At this step, *R*^2^ = 0.17 was statistically significant, *F*_(1,71)_ = 14.05, *p* < 0.001. After the entry of the three measures of executive functioning (Stroop, TMT, and SOPT) at step 2, the total variance explained by the model as a whole was 22.4%, *F*_(4,68)_ = 4.91, *p* < 0.01. The three measures of executive functioning explained an additional 5.9% of the variance in the amplitude of the parietal positivity for the central electrode region after controlling for Age, *R*^2^ change = 0.06, *F* change (3,68) = 1.72, *p* = 0.17. Although the overall *R* change at this step was not statistically significant, SOPT (beta = −0.22, *p* < 0.05) was found to be a significant predictor of the amplitude of the parietal positivity for the central electrode region.

##### Right electrode region

Age was entered at step 1, explaining 7% of the variance in the amplitude of the parietal positivity. At this step, *R*^2^ = 0.07 was statistically significant, *F*_(1,71)_ = 5.32, *p* < 0.05. After the entry of the three measures of executive functioning (Stroop, TMT, and SOPT) at step 2, the total variance explained by the model as a whole was 16.4%, *F*_(4,68)_ = 3.34, *p* < 0.05. The three measures of executive functioning explained an additional 9.5% of the variance in the amplitude of the parietal positivity for the right electrode region after controlling for Age, *R*^2^ change = 0.10, *F* change (3,68) = 2.56, *p* = 0.06. Although the overall *R* change at this step was only marginally statistically significant, SOPT (beta = −0.29, *p* < 0.05) was found to be a significant predictor of the amplitude of the parietal positivity for the right electrode region.

##### Left electrode region

Age was entered at step 1, explaining 0.10% of the variance in the amplitude of the parietal positivity. At this step, *R*^2^ = 0.001 was not statistically significant, *F*_(1,71)_ = 0.06, *p* = 0.80. After the entry of the three measures of executive functioning (Stroop, TMT, and SOPT) at step 2, the total variance explained by the model as a whole was 2.9%, *F*_(4,68)_ = 0.51, *p* = 0.73, The *R*^2^ change = 0.03 was not statistically significant, *F* change (3,68) = 0.66, *p* = 0.58.

## Discussion

The aim of the current study was to examine the neural correlates of PM (i.e., the N300 and parietal positivity) across adolescence. First, if processes underlying the prospective component of PM continue to develop across adolescence, then the amplitude of the N300 should reveal an age-related difference across the age groups (Wang et al., 2011). Conversely, if processes underlying the retrospective component of PM continue to develop across adolescence, then the amplitude of the parietal positivity should reveal an age-related difference across the age groups (Zöllig et al., 2007). Finally, it was expected that executive functions (viz., working memory, inhibition, and task-switching) would be important in predicting the amplitude of the ERP components of PM. More specifically, if nonfocal PM tasks place higher demand on executive control processes needed to detect PM cues (e.g., Ward et al., 2005; Shum et al., 2008; Altgassen et al., 2014), then executive functioning may have a greater contribution to the amplitude of N300 (i.e., prospective component of PM). Conversely, it was unclear as to the extent that executive functioning would contribute to the amplitude of parietal positivity. In particular, if this component encompasses both the prospective and retrospective component of PM (West, 2011), then executive functions may also contribute to the amplitude of this component. The study was exploratory in nature and was the first to explore how executive functions may contribute to the neurocognitive processes that underlie PM in adolescence.

First, PM performance (percentage correct) was found to be similar across the age groups on a task that utilized nonfocal cues. On the other hand, the PM composite score (i.e., combined score of percentage correct and RT for percentage correct) indicated that 12 to 13-year-olds had poorer PM performance than the 18 to 19-year-olds. This finding suggests that there may have been a speed/accuracy trade-off in PM performance across the age groups. In particular, when considering only percentage correct scores, no age-related effect was apparent. Taken together, this finding partially supports the proposal that age-related differences in PM should emerge on a task that loads heavily on executive control processes (i.e., nonfocal cues, Wang et al., 2011; Altgassen et al., 2014). One explanation for the findings here could be that both executive control and nonexecutive processes may have contributed to the likelihood of age-related differences in PM emerging. In particular, previous studies have suggested that processing speed continues to develop across adolescence (e.g., Luna et al., 2004). In fact, the overall improvement of processing speed has been linked to increased efficiency in the recruitment of cognitive networks associated with the task (Spear, 2000). Moreover, the use of more efficient cognitive strategies (e.g., slowing down on trials to improve accuracy) may have been advantageous for adolescents in maintaining a high degree of PM accuracy (Davidson et al., 2006). This highlights the importance in taking RT into account when examining PM performance across different age groups (Kvavilashvili et al., 2008). Specifically, taking into account RT on PM performance may have increased the sensitivity in detecting age-differences on the PM task than when examining PM percentage correct scores alone (Best and Miller, 2010).

Second, for the electrophysiological findings, the overall mean amplitude of the N300 was found to be similar across the age groups. In contrast, the mean amplitude for the parietal positivity was found to decrease with age, with the difference being significant between the 12 to 13-year-olds and the 18 to 19-year-olds for PM trials. In addition, the findings showed that the age-related difference in the parietal positivity was specifically marked in the central and right electrode regions of the brain, but not the left electrode brain region. Taken together, this pattern of results is consistent with the findings of Zöllig et al. (2007), which suggested that processes underlying the retrospective component of PM (i.e., parietal positivity) might continue to develop across adolescence. It is important to note, however, that the parietal positivity may encompass processes that are associated with both the prospective component of PM (i.e., detection of PM cues) and the retrospective component of PM (retrieval of intention from memory). In addition, some studies suggest that there may be an age-related difference in the neural recruitment of PM processes supporting the retrospective component of PM from childhood to young adulthood (e.g., Zöllig et al., 2007; Mattli et al., 2011). In particular, this age-related difference in neural recruitment may reflect the differential maturation of the prefrontal lobes from childhood to adulthood (Segalowitz and Davies, 2004; Blakemore and Choudhury, 2006).

Some caution needs to be taken in regards to interpreting both the behavioral and ERP data. First, the effect size for the N300 (in regards to finding age differences) may be smaller and harder to detect; therefore, a greater number of participants may be needed to find an age effect on this component. Second, the decrease in the amplitude of the parietal positivity across adolescence may be due to age-related developmental differences in one of the subcomponents of the parietal positivity (i.e., P3b, recognition old-new effect, or prospective positivity; Mattli et al., 2011; West, 2011). In fact, research has shown that the amplitude of the P3b (viz., the visual P3b) decreases across childhood and adolescence (e.g., Houston et al., 2005). This developmental change has been attributed to the fine-tuning of cognitive networks that occur during adolescence (Segalowitz et al., 2010). Moreover, the P3b has been found to show a latency and topographic shift with age (i.e., peaks become more focal and later in latency with age; Segalowitz and Davies, 2004; Segalowitz et al., 2010). Segalowitz et al. (2010) proposed that this change might reflect a deeper processing of the target cue. Therefore, children and adolescents may take longer to respond to the targets on given trials. While this proposal fits well with the behavioral findings of this study, more research is needed to determine the specific processes that contribute to PM development in adolescence.

Finally, in regards to the contribution of executive functioning measures on the neural correlates of PM, no measures of executive functioning were found to predict the amplitude of the N300, after controlling for age. In contrast, a measure of visual working memory (viz., SOPT) was found to predict the amplitude of the parietal positivity (for right and central electrode regions only), after controlling for age. This finding suggests that visual working memory abilities contributed to neurocognitive processes that underlie the parietal positivity. Importantly, one possible explanation for this may have to do with the perceptual nature of the PM cue (color). In particular, working memory processes are believed to be associated with keeping the PM cue in mind (refreshing or updating the status of the PM cue) while working on the ongoing task (Kliegel et al., 2008). Furthermore, based on their LORETA findings, Zöllig et al. (2007) found evidence that suggested that adolescents had greater activation in brain regions associated with visual imagery (viz., precuneus region) compared to young adults. The precuneus region is assumed to be associated with the maintenance of the PM response or rehearsal of the target stimuli (see Burgess et al., 2001). Therefore, the findings of the current study may suggest that working memory processes (which may have taken form as monitoring processes) contributed to successful PM performance on this task (McDaniel and Einstein, 2000; Smith, 2003). In addition, it may also indicate that executive control processes may contribute to the neurocognitive processes that underlie the parietal positivity. In contrast, inhibition (as measured by the Stroop task) and task-switching (as measured by TMT) did not appear to contribute to the ERP components of PM. One possible explanation for this may pertain to the characteristics of the PM task used in this study. Specifically, the number of PM cues used in this study was high in frequency (30 cues) and repetition. PM cues that are high in frequency and repetition may be easier to monitor than for cues that are low in frequency and repetition (Wilson et al., 2013). Therefore, the current PM paradigm used in this study may have placed low demands on executive functions associated with switching between the ongoing and PM component of the task, particularly when the PM cue had been encountered a number of times.

Taken together, the findings of this study suggest that PM may still be developing in adolescence. Some limitations of this study need addressing. First, ceiling effects could have masked the true age-related effect of PM across adolescence when percentage correct scores are considered. That said, the PM composite score showed that there were age-related differences in PM across adolescence. Moreover, for EEG research, a maximal number of correct trials are needed in order to obtain good averaged ERPs (Luck, 2005). Therefore, the aim of the study was to design a paradigm that was equivalent across different age groups (in terms of cognitive demand), but sensitive enough to detect subtle age differences (Kvavilashvili et al., 2008).

Second, ongoing task performance (ongoing task composite score) was found to be the lowest for 12 to 13-year-olds and the 14 to 15-year-olds than the 18 to 19-year-olds. Importantly, Kvavilashvili et al. (2008) argued that if both the ongoing task and the embedded PM task compete for attentional resources, age-related effects in PM may be distorted or masked. While accuracy on the ongoing component was over 90% for across age groups, there may have been a trade-off in accuracy between the ongoing and PM task components. Although this seems unlikely given the high accuracy rate, research should address the issue of ongoing task difficulty, especially for ERP studies (where use longer duration tasks are used).

Third, this study may have been slightly underpowered. For instance, for an 80% chance of detecting a relationship (if in fact one did exist), a total of 77 participants would be needed in order to detect a medium effect size. The total sample size for the current study was 75 participants (there was an attrition of 10 participants’ EEG recordings). Therefore, the current study may have been slightly underpowered in the ability to detect significant relationship (especially for the N300). Despite this, the task was sensitive enough to detect significant effects for the mean amplitude of parietal positivity. Even though most ERP studies utilize smaller sample sizes, future research should examine the role of executive functioning on the neural correlates of PM in larger samples.

In conclusion, the findings of this study suggest that the PM may still be developing in adolescence (Zöllig et al., 2007). In addition, this is the first study to link executive functioning (viz., visual working memory) to the neural correlates of PM (viz., the parietal positivity). The findings of this study may help inform specific models of PM (i.e., MPT and PAM theory). It may also provide further insight into how PM develops across adolescence. Finally, the findings of this study may help inform diagnostic practices for adolescent and adult psychopathology (e.g., Hill et al., 1999).

## Conflict of Interest Statement

The authors declare that the research was conducted in the absence of any commercial or financial relationships that could be construed as a potential conflict of interest.

## Footnotes

^1^There was a technical issue with the randomization of the PM cues for the first 21 participants. The cues were not appearing randomly (viz., it appeared on every 20th trial). This was corrected as soon as it was noticed. No notable differences were found between the means of the first 21 participants and the following participants. Thus, the first 21 participants were retained in the study.

^2^Note. Only the PM task included an EEG component.
